# Role of Spinal Cord Akt-mTOR Signaling Pathways in Postoperative Hyperalgesia Induced by Plantar Incision in Mice

**DOI:** 10.3389/fnins.2020.00766

**Published:** 2020-07-22

**Authors:** Bing Xu, Su-Su Liu, Jin Wei, Zi-Yin Jiao, Cheng Mo, Cheng-Mei Lv, Ai-Lan Huang, Qi-Bo Chen, Li Ma, Xue-Hai Guan

**Affiliations:** ^1^Department of Rehabilitation, The People’s Hospital of Guangxi Zhuang Autonomous Region, Nanning, China; ^2^Department of Anesthesiology, The People’s Hospital of Guangxi Zhuang Autonomous Region, Nanning, China; ^3^Department of Anesthesiology, The First Affiliated Hospital of Guangxi Medical University, Nanning, China

**Keywords:** the mammalian target of rapamycin kinase, protein kinase B, incisional pain, spinal dorsal corn, mice

## Abstract

Poor postoperative pain (POP) control increases perioperative morbidity, prolongs hospitalization days, and causes chronic pain. However, the specific mechanism(s) underlying POP is unclear and the identification of optimal perioperative treatment remains elusive. Akt and mammalian target of rapamycin (mTOR) are expressed in the spinal cord, dorsal root ganglion, and sensory axons. In this study, we explored the role of Akt and mTOR in pain-related behaviors induced by plantar incision in mice. Plantar incision activated spinal Akt and mTOR in a dose-dependent manner. Pre-treatment with Akt inhibitors intrathecally prevented the activation of mTOR dose-dependently. In addition, blocking the Akt-mTOR signaling cascade attenuated pain-related behaviors and spinal Fos protein expression induced by plantar incision. Our observations demonstrate that Akt-mTOR might be a potential therapeutic target for the treatment of POP.

## Introduction

Surgery is a first-line treatment option for many diseases and can be associated with numerous complications including, but not limited to, acute postoperative pain (POP). Poor POP control increases perioperative sickness, prolongs hospitalization days, and causes chronic pain (Reddi and Curran, 2014), all of which incur a significant cost to society in terms of subsequent healthcare costs (Turk, 2002). Patients often experience pain as a result of surgical trauma and it is difficult to reduce perioperative opioid use. Unfortunately, opioids are often abused and can lead to poor health outcomes and an increased risk of mortality. Therefore, in an attempt to reduce opioid use, it is necessary to investigate and utilize non-opioid agents to provide adequate pain management.

Recent studies indicate that activation of the serine/threonine protein kinase, Akt (also known as PKB), contributes to pain-related behaviors induced by various kinds of stimuli, including capsaicin, BDNF, and chronic nerve constriction (Sun et al., 2006, 2007; Xu et al., 2007, 2011; Guedes et al., 2008; Choi et al., 2012; Duan et al., 2012; Kay et al., 2013; Li et al., 2013; Hung et al., 2014; Xu B. et al., 2014; Zhang et al., 2014). Akt plays an important role in various key biological functions such as cell cycle processes, apoptosis, proliferation, survival, and growth in response to cytokines or growth factors (Manning and Cantley, 2007; Yang et al., 2011). Importantly, Akt is widely expressed in the spinal cord, particularly in the dorsal root ganglia (DRG) and laminae I-IV of the spinal dorsal horn, a key site for processing nociceptive information (Sun et al., 2006, 2007; Xu et al., 2007, 2011; Guedes et al., 2008; Choi et al., 2012; Duan et al., 2012; Kim et al., 2012; Li et al., 2013; Kay et al., 2013; Hung et al., 2014; Xu J.T. et al., 2014; Zhang et al., 2014). The role of Akt in acute POP remains elusive.

The mammalian target of rapamycin kinase (mTOR), a downstream signaling molecule of Akt, regulates transcription, initiation of translation, ribosome biosynthesis, and apoptosis (Hay and Sonenberg, 2004). In addition, mTOR is a master regulator of protein synthesis in neuronal axons and dendrites (Takei et al., 2004) and is critically involved in the regulation of neuronal functions that mediate memory formation and synaptic plasticity within the central nervous system (Hoeffer and Klann, 2010; Lyu et al., 2013). Recent evidence indicates that mTOR is expressed within the spinal cord and in sensory axons (Geranton et al., 2009; Obara et al., 2011; Cui et al., 2014) and that blocking of mTOR alleviated pain-related behaviors associated with local inflammation or neuropathic pain (Jimenez-Diaz et al., 2008; Geranton et al., 2009). Given that both trauma and inflammatory factors are involved in POP and that Akt plays a role in synaptic plasticity and memory (through the regulation of mTOR) (Klann and Dever, 2004; Costa-Mattioli et al., 2009) we explored whether Akt-related signaling may play an important role in POP. To this end, the following experiments were designed to examine the activation pattern and cellular expression of spinal Akt and further characterized its involvement in POP by using pharmacological inhibitors of the Akt pathway. Additionally, we confirmed whether mTOR was necessary to initiate the analgesic effects of Akt inhibitors. Our results demonstrated that Akt mediates pain-related behaviors induced by plantar incision via activation of the mTOR pathway. Moreover, these data suggest that pharmacological inhibition of Akt-mTOR could be a potential strategy to alleviate POP.

## Materials and Methods

### Animals and Ethics Statement

Eight-week-old male Kunming mice, weighing between 20 and 25 g (*N* = 288; Guangxi Medical University, Nanning, PR China), were housed under a 12 h/12 h light-dark cycle regime at a constant temperature of 22°C with *ad libitum* access to food and water. All experimental protocols were approved by the local Animal Care and Use Committee (The People’s Hospital of Guangxi Zhuang Autonomous Region, Nanning; No. 2018-06) and were in accordance with the Declaration of the National Institutes of Health Guide for Care and Use of Laboratory Animals (Revised 2011). Mice were randomly allocated to the different experimental groups. Measures were taken to minimize the pain and discomfort of the experimental animals.

### Drug Preparation and Administration

For intrathecal injection, all agents were prepared to be delivered in 5 μl. The Akt inhibitors (Akt IV, Merck, Cat No. 124011; MK2206, Selleck, Cat No. S1078, Lot No. 06) and mTOR inhibitors [Ridaforolimus (Rida), Selleck, Cat No. S1022; Rapamycin (Rapa), Gene Operation, Cat No. IPA1021-0050MG, Lot No. QRB1701] were dissolved in 5% dimethyl sulfoxide (DMSO) in physiological saline. Control mice were injected with 5% DMSO in physiological saline (vehicle). Drug doses were based on preliminary experiments. The doses and treatment timeline are presented in the figure legends and “Results” section.

### Intrathecal Drug Administration

Intrathecal drug administration was done as previously described by Hylden and Wilcox (1980). Briefly, a 28-gauge stainless steel needle attached to a 25 μl Hamilton micro syringe was inserted between the L6 and L5 vertebrae in conscious animals. Correct position of needle tip was confirmed by observation of a sudden tail flick. The drugs or vehicle were injected within 30 s, into the subarachnoid space and the needle tip was left in place for an additional 15 s. Mice with any signs of motor dysfunction were excluded from this study.

### Plantar Incision Model

Plantar incision was performed according to the method previously described by Pogatzki and Raja (2003). Mice were anesthetized with 1.5–2% aerosolized isoflurane delivered via a nose cone. The incision site was sterilized using a 10% povidone-iodine solution and using a No. 11 blade, a 5 mm longitudinal incision was made through the skin and fascia of the plantar aspect of the right paw, starting 2 mm from the proximal edge of the heel and extending toward the digits. The flexor digitorum brevis muscle was elevated and incised, while the muscle origin and insertion remained intact. The skin was stitched together with a single mattress suture of 8-0 nylon on a TG175-8 needle. The wound site was treated with antibiotic ointment.

### Behavioral Testing

All behavioral measurements were recorded by observers blinded to the treatment groups. In order to minimize stress and exploratory activity, all animals were acclimated to the testing room for 1–2 days prior to starting the experiment. Mechanical allodynia was evaluated by using von Frey filaments (Stoelting, IL), starting with 0.07 g and ending with 2.0 g in ascending order, as previously described (Pogatzki and Raja, 2003; Xu B. et al., 2014). Mice were placed in individual plastic boxes (5 × 5 × 8) with a metal mesh floor and allowed to acclimate for 30 min. The tips of filaments were perpendicularly applied to the plantar surface, with sufficient force to cause slight bending against the paw, and held for 1 s. Each filament was applied 5 times in 10 s intervals. Paw flinching or brisk withdrawal was considered a response. Paw withdrawal frequency (PWF) to each monofilament was calculated and paw withdrawal threshold (PWT) was considered the force at which PWF ≥ 60%; and 2 g was used as the PWT if all PWF < 60%.

According to the method described by Hargreaves et al. (1988), the paw withdrawal latency from a radiant heat machine, was used to measure thermal hyperalgesia. Briefly, mice were acclimated to the test chamber (5 × 5 × 8) for 1 h on a glass platform at 25°C. The radiant heat generated by a halogen projection bulb was focused on the area of the ipsilateral paw from the underneath the glass. The time required to cause an abrupt withdrawal of the ipsilateral paw was regarded as the PWL. A cutoff of 20 s was used to avoided tissue damage. This was repeated three times at 5 min intervals and the three values were averaged to provide the final PWL for each mouse.

A cumulative pain score (CPS) was obtained based on the position of the ipsilateral paw during the majority of a 60 s scoring period, with observations taken at 5 min intervals over 1 h (Brennan et al., 1996). Mice were placed in individual plastic boxes (5 × 5 × 8) with a metal mesh floor (grid 5 × 5 mm) and allowed to acclimate for 60 min. A score of 0 was given if the paw was distorted or blanched by the mesh; 1, if the paw touched the mesh without distorting or blanching; and 2, if the paw was completely off the mesh.

AUC (from −4 to 48 h) value of PWT, PWL and CPS was calculated for each animal separately, and the average value of 10 animals represented the final AUC of this group.

### Immunohistochemistry

Mice were deeply anesthetized with 2% isoflurane delivered via a nose cone and subsequently transcardially perfused with 30 ml of saline, followed by 100 ml of ice-cold paraformaldehyde (4%) in phosphate-buffered saline (PBS). The L3-5 segments of the spinal cord were dissected and post-fixed in 4% paraformaldehyde overnight at 4°C, and were then transferred into 30% sucrose in PBS overnight at 4°C. Twenty micrometer transverse sections were cut on a cryostat (Leica) and every fifth section was mounted on gelatin-coated slides. After washing with PBS, the sections were penetrated with 0.3% Triton X-100 at room temperature (RT) for 15 min. Non-specific binding was blocked by incubation in 5% normal goat serum in PBS at RT for 30 min. Sections were then incubated in primary polyclonal rabbit-anti-Fos antibody (a biologic marker of neuronal activation, 1:1000, Cell signaling Technology, RRID:AB 2247211) at 4°C for 24 h. After washing with PBS (3 × 5 min), the sections were incubated in a biotinylated goat-anti-rabbit (1:200, ZSGB-Bio, RRID:AB_2758396) secondary antibody at 37°C for 1 h, followed by incubation in avidin-biotin-peroxidase complex (1:100, ZSGB-Bio, RRID:AB_2758396) at 37°C for 2 h. The sections were then washed with PBS before being treated with 0.05% diaminobenzidine for 5–10 min. Finally, the sections were rinsed in PBS to stop the reaction, air-dried, dehydrated with 70–100% alcohol, cleared with xylene and then cover-slipped for microscopic examination.

To analyze alterations of Fos protein levels, every fifth section was selected from a series of consecutive sections for each mouse, and the total number of Fos protein in the ipsilateral lamina (I-IV) were counted. All positive cells were counted without considering the staining intensity. The average number in each section represented the change of Fos protein. All data were expressed as mean ± SEM.

For immunofluorescence staining, sections were incubated in the following primary antibodies: monoclonal rabbit-anti pmTOR (1:50, Abcam, RRID:AB_10888105) and a monoclonal anti-neuronal nuclei (NEUN; neuronal marker, 1:200; Millipore, RRID:AB_2298772), monoclonal anti-glial fibrillary acidic protein (GFAP; astrocytes marker, 1:400; CST, RRID:AB_561049); or polyclonal anti-IBA1 (IBA1; microglia marker, 1:300; Abcam, RRID:AB_2224402). All sections were incubated in their respective primary antibodies at 4°C for 48 h. After washing with PBS (3 × 5 min), the sections were treated with a mixture of CY3-conjugated immunoglobulin G (1:500, Abcam), Alexa Fluor 488-conjugated immunoglobulin G (1:200) secondary antibodies, at 37°C for 2 h. For nuclear staining, 4′6-diamidino-2-phenylindole (DAPI; 500 ng/ml; Thermo Fisher Scientific, RRID:AB_2629482) was used in the last 10 min before a final PBS wash (3 × 5 min). Non-specific labeling was determined running the same protocol but excluding incubation with the primary antibodies. Sections were then mounted and cover-slipped with 75% glycerol and stored at −20°C in the dark. Images were taken using a fluorescence microscope (BX51, Olympus).

### Western Blot

Mice were deeply anesthetized by 2% isoflurane and decapitated. The whole spinal cord at L3-5 segments was hydro-extruded from the vertebral column using a 5 ml syringe filled with an ice-cold 0.9% saline solution and stored in liquid nitrogen. Samples were homogenized in extraction buffer containing the following (in mM): Tris, 20.0, pH 7.4; Sucrose, 250; Na_3_VO_4_, 0.03; MgCl_2_, 2.0; EDTA, 2.0; EGTA, 2.0; phenylmethylsufonyl fluoride, 2.0; dithiothreitol, 1.0; protease inhibitor cocktail, 0.02% (v/v). The homogenized samples were centrifuged at 12,000 g for 30 min at 4°C. The supernatant was collected and the concentration was determined using a BCA protein assay kit. Each sample was dissolved in 5× sample buffer and denatured at 95°C for 5 min. Samples containing equal weights of protein (30 μg) were separated by using a gradient (4–8%) SDS-PAGE gel and transferred onto a polyvinylidene fluoride membrane. All membranes were blocked in 5% bovine serum albumin for 2 h at RT and were then incubated with the following primary antibodies for 24 h at 4°C: rabbit anti-mTOR (1:1000, Abcam, RRID:AB_881283), rabbit anti-phospho mTOR (pmTOR, 1:1000, Abcam, RRID:AB_10888105), or rabbit anti β-actin (1:5000, Bioss, RRID:AB_10855480) made in the blocking solution. After washing with tris-buffered saline with Tween-20 three times (5 min each time), the membranes were incubated with a secondary antibody conjugated with horseradish peroxidase (1:3000, CST, RRID:AB_2099233) for 2 h at RT. The membranes were then incubated with electrogenerated chemiluminescence for 2 min and exposed in Odyssey Fc Image System (LI-COR) for 1–10 min. Gray value of each blot were calculated by Image Studio Ver 5.2. The gray value of pmTOR was normalized to a house keeping protein (β-actin) followed by normalization to its corresponding mTOR band. All mTOR bands were normalized to β-actin. The blot density for control mice was set as 1.

### Co-immunoprecipitation Analysis

Total protein extracts were prepared from the spinal cord at the L3-5 segments (4 h after plantar incision or sham operation) using ice-cold immunoprecipitation buffer (Beyotime) containing 0.1 mM phenylmethylsulfonyl fluoride protease inhibitor and a protease inhibitor cocktail (0.02%, v/v; CST). The homogenized samples were centrifuged at 12000 g for 10 min at 4°C. After incubating with rabbit anti-pmTOR (10 μg, Abcam, RRID:AB_10888105) overnight at 4°C, the protein extracts (500 μg) were incubated with protein A/G agarose (Calbiochem, IP05) on a rotator for 3 h. In the negative control group, normal rabbit IgG (2 μg, CST, RRID:AB_1031062) was used in place of anti-pmTOR. After washing with immunoprecipitation buffer five times, the mixture was dissolved in 2× sample buffer and denatured at 95°C for 5 min. Samples containing equal weights of protein (30 μg) or total protein extract (10 μg, as a positive control, input) were detected by western blotting.

### Statistical Analysis

All data were expressed as mean ± SEM, and were analyzed using GraphPad Prism 5.01 software. One-way analysis of variance (ANVOA) or two-way ANVOA with Bonferroni post tests were used where appropriate. “Drug” was treated as a “between” subject factor. “Time” was treated as “within subjects” factor. Differences with *P-*values of less than 0.05 were considered statistically significant.

## Results

### Blocking of Spinal Akt Prevented Pain-Related Behavior Induced by Plantar Incision

**FIGURE 1:**
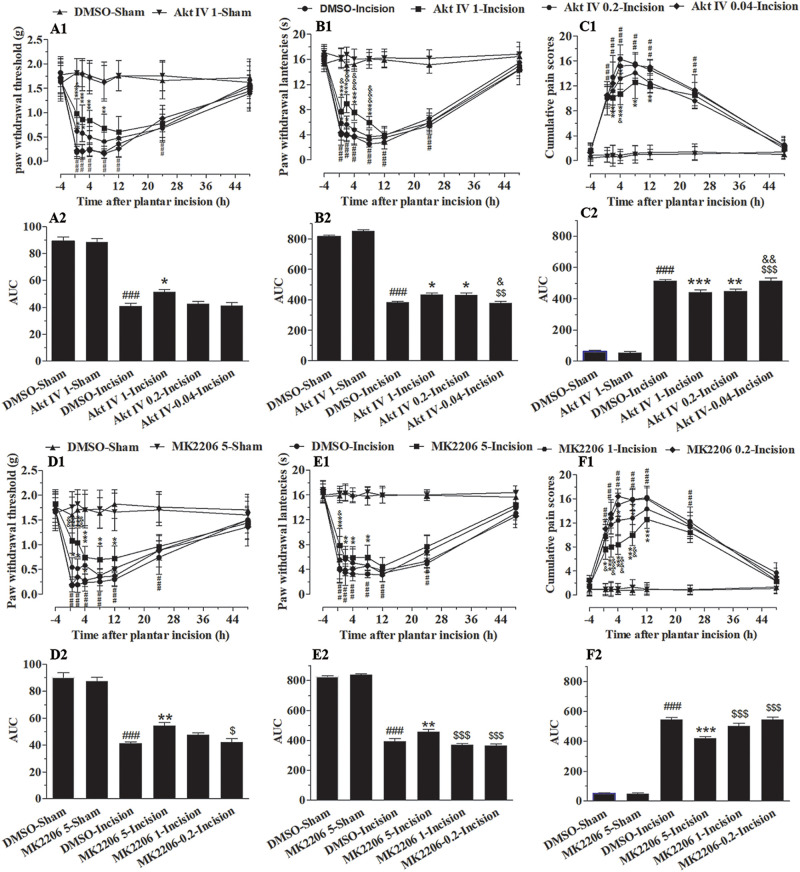
Blocking of spinal Akt attenuated mechanical allodynia, thermal hyperalgesia, and cumulative pain scores induced by plantar incision. Two selective inhibitors of Akt, Akt IV (0.04, 0.2, and 1 μg in 5% DMSO 5 μl), MK2206 (0.2, 1, and 5 μg in 5% DMSO 5 μl), or vehicle (5% DMSO 5 μl) were intrathecally injected 30 min before plantar incision. PWT to mechanical stimuli, PWL to radiant heat and CPS were recorded at −4, 0.5, 2, 4, 8, 12, 24, and 48 h after plantar incision. Pre-treatment with various doses of Akt IV **(A1–C1)** or MK2206 **(D1–F1)** attenuated the decrease of PWT **(A1,D1)** and PWL **(B1,E1)**, or the increase of CPS **(C1,F1)** induced by plantar incision. The calculated area under the curve (AUC) was significantly increased in Akt IV 1- or MK2206 5-Incision groups in PWL **(B2,E2)** and PWT **(A2,D2)** tests, and was significantly decreased in Akt IV 1-, Akt IV 0. 2-, or MK2206 5-Incision groups in CPS **(C2,F2)** tests. ^###^*P*<0.001, compared with DMSO-Sham group; ^∗^*P*< 0.05, ^∗∗^*P* < 0.01, ^∗∗∗^*P*< 0.001, compared with the DMSO-Incision group;^&^*P*<0.05, ^&⁣&^*P*<0.01, ^&⁣&⁣&^*P*<0.001, compared with the Akt IV 0.2-Incision group or MK2206 1-Incision group; ^$^*P*<0.05, ^$$^*P*<0.01, ^[*dollar*][*dollar*][*dollar*]^P < 0.001. compared with Akt IV 1-Incision group or MK2206 5-Incision group. **(A1–F1)** Two-way repeated measure ANOVA were applied to all comparisons, followed by Bonferroni’s posttest. “Drug” was treated as a “between” subject factor, “Time” was treated as “within subjects” factor. **(A1)** Drug: *F*(5, 432) = 247.96, *P*< 0.0001; Time: *F*(7, 432) = 86.53, *P*< 0.0001; **(B1)** Drug: *F*(4, 315) = 726.26, *P*< 0.0001; Time: *F*(7, 315) = 430.04, *P*< 0.0001; **(C1)** Drug: *F*(4, 315) = 366.06, *P*< 0.0001; Time: *F*(7, 315) = 286.03, *P*< 0.0001; **(D1)** Drug: *F*(7, 315) = 123.9, *P*< 0.0001; Time: *F*(4, 315) = 149.59, *P*< 0.0001. **(E1)** Drug: *F*(7, 315) = 266.95, *P*< 0.0001; Time: *F*(4, 315) = 468.07, *P*< 0.0001. **(F1)** Drug: *F*(7, 315) = 288.19, *P*< 0.0001; Time: *F*(4, 315) = 471.47, *P*< 0.0001. **(A2–F2)** One-way ANOVA followed by Bonferroni’s Multiple Comparison Test. *n* = 10. Data were presented as mean ± SEM.

To define the involvement of spinal Akt in plantar-induced pain-related behavior, we examined the capacity of intrathecal delivery of Akt inhibitors to attenuate pain induced by plantar incision. Two selective inhibitors of Akt, Akt IV (0.04, 0.2, and 1 μg in 5 μl of 5% DMSO) and MK2206 (0.2, 1, and 5 μg in 5 μl of 5% DMSO), were administered intrathecally 30 min before plantar incision. The pre-treatment of Akt IV and MK2206 attenuated mechanical allodynia and thermal hyperalgesia in a dose-dependent manner (Figure 1). Compared to mice treated with DMSO, PWL was significantly elevated by Akt IV treatment (Figure 1B1: 0.2 μg at 0.5–2 h; 1 μg at 0.5–8 h) and by MK2206 treatment (Figure 1E1: 1 μg at 0.5–4 h; 5 μg at 0.5–12 h) (*P*< 0.05). Compared to mice treated with DMSO, PWT was significantly elevated by Akt IV treatment (Figure 1A1: 0.2 μg at 0.5–2 h; 1 μg at 0.5–8 h) and by MK2206 treatment (Figure 1D1: 1 μg at 2 h; 5 μg at 0.5–8 h) (*P*< 0.05). CPS also differed between treatment- and vehicle-treated mice. CPS was significantly decreased by Akt IV treatment (Figure 1C1: 0.2 μg at 2–4 h; 1 μg at 2–12 h) and by MK2206 treatment (Figure 1F1: 1 μg at 4–8 h; 5 μg at 2–12 h) (*P*< 0.05). Behavioral assessment revealed that intrathecal Akt IV or MK2206 did not alter the PWL, PWT, or CPS in Sham-surgerized mice. Similarly, intrathecal vehicle (5 μl of 5% DMSO) did not affect pain-related behavior at any time points. The calculated area under the curve (AUC) was significantly increased in Akt IV 1- or MK2206 5-Incision groups in PWL (Figures 1B2,E2) and PWT (Figures 1A2,D2) tests (*P*< 0.05 or *P*< 0.01, respectively), and was significantly decreased in Akt IV 1-, Akt IV 0. 2-, or MK2206 5-Incision groups in CPS (Figures 1C2,F2) tests (*P*< 0.05 or *P*< 0.01, respectively).

### Inhibition of Spinal Akt Attenuated the Up-Regulation of Spinal Fos Protein Induced by Plantar Incision

**FIGURE 2:**
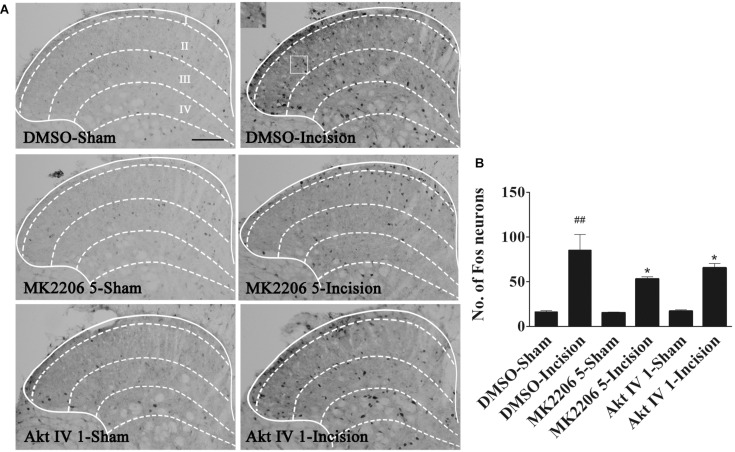
Blocking of spinal Akt prevented the up-regulation of spinal Fos protein expression induced by plantar incision. Inhibitors of Akt, Akt IV (1 μg in 5% DMSO 5 μl), MK2206 (5 μg in 5% DMSO 5 μl), or vehicle (5% DMSO 5 μl) were intrathecally injected 30 min before plantar incision. Fos protein expression was assayed at 1 h after plantar incision. **(A)** Representative immunohistochemical staining of spinal Fos protein expression. **(B)** Quantitative analysis of spinal Fos protein expression.^##^*P*<0.01, compared with DMSO-Sham group; *P<0.05, compared with DMSO-Incision group. One-way ANOVA followed by Bonferroni’s Multiple Comparison Test. *n* = 6, scale bar = 200 μm.

Spinal central sensitization was involved in the development and maintenance of pain. Fos protein was used as a biologic marker of neuronal activation (Coggeshall, 2005; Guan et al., 2010; Xu J.T. et al., 2014). In order to clarify the analgesic effect of Akt inhibitors, Akt IV (1 μg in 5 μl of 5% DMSO), MK2206 (5 μg in 5 μl of 5% DMSO), or 5% DMSO (5 μl) were intrathecally injected 30 min before plantar incision. Fos expression 1 h after plantar incision was evaluated by immunohistochemistry. We observed that spinal Fos immunoreactivity was expressed in ipsilateral laminae I–IV (Figure 2), suggesting that the spinal neurons could be activated by plantar incision. Compared to the DMSO-sham group, plantar incision produced a significant increase of Fos protein in the ipsilateral spinal dorsal horn of the DMSO-Incision group (Figures 2A,B). Compared to the DMSO-Incision group, Akt IV and MK2206 significantly attenuated the increase of Fos protein in the Akt IV 1- and the MK2206 5-group (*P*< 0.05). Intrathecal vehicle (5% DMSO), Akt IV, or MK2206 did not alter the Fos expression in sham-surgery mice.

### Plantar Incision Induced a Time-Dependent Activation of mTOR in Spinal Cord

**FIGURE 3:**
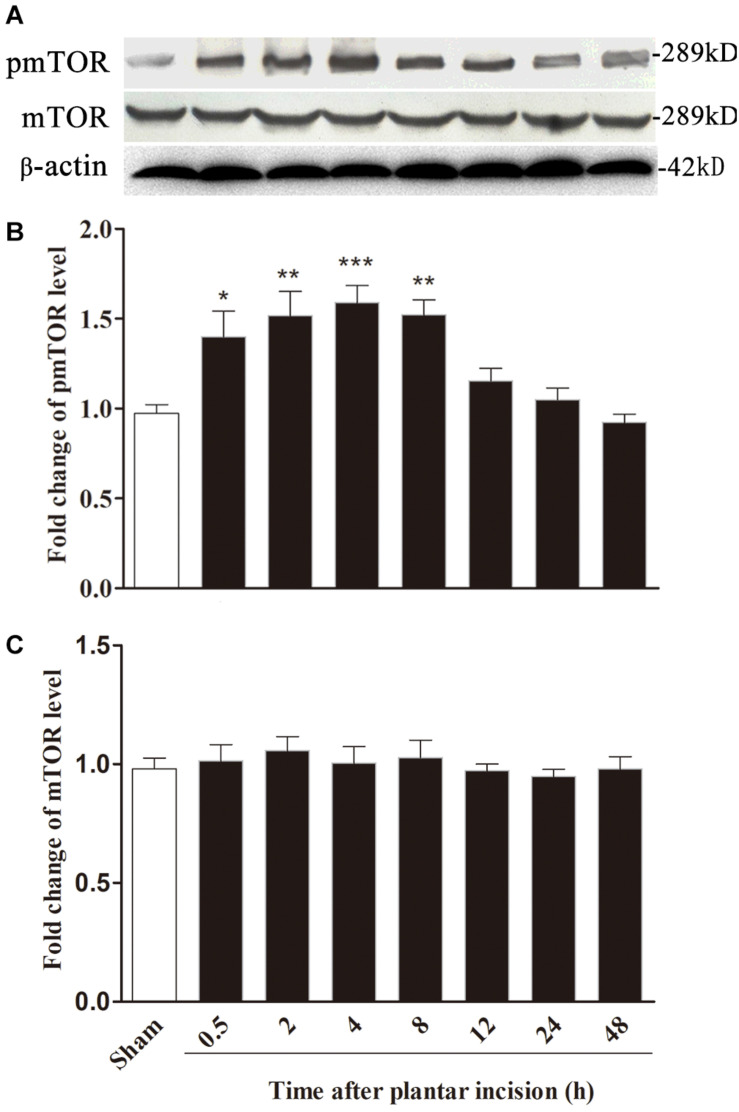
Plantar incision induced a time-dependent activation of mTOR in ipsilateral spinal cord. The expression of pmTOR and mTOR was assayed at sham, 0.5, 2, 4, 8, 12, 24, and 48 h after plantar incision. Representative bands **(A)** of protein expression at different time point after plantar incision and the quantitative data of pmTOR **(B)** and mTOR **(C)** expression are shown. *P<0.05, **P<0.01, ***P<0.001, compared with sham group. One-way ANOVA followed by Dunnett’s Multiple Comparison Test. *n* = 4. Data were presented as mean ± SEM.

In parallel with the time course of the development of hyperalgesia, plantar incision induced an increase in mTOR phosphorylation (pmTOR) in the ipsilateral side of the spinal cord dorsal horn, suggesting that mTOR is activated in response to plantar incision. Compared to sham-surgerized controls, pmTOR increase started at 0.5 h, reached peak expression at 4 h, and returned to baseline 24 h after plantar incision (Figures 3A,B). Expression of mTOR on the ipsilateral side of the dorsal horn did not change significantly between incised and sham controls (Figures 3A,C). Expression of pmTOR on the contralateral side of the dorsal horn did not change significantly between incised and sham controls (data was not shown).

### Distribution and Cellular Localization of Spinal Activated mTOR

**FIGURE 4:**
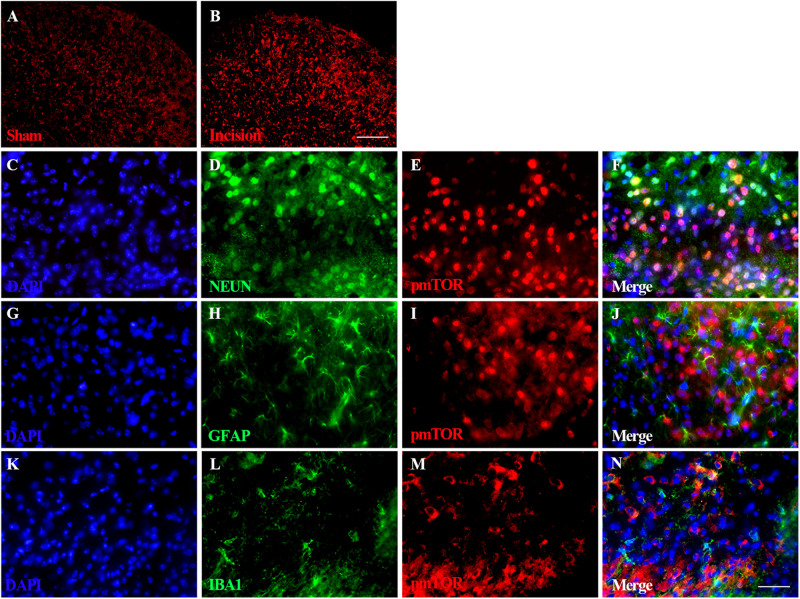
Distribution and cellular localization of spinal activated mTOR. Immunofluorescence staining of spinal pmTOR was performed at 4 h after plantar incision. Triple immunofluorescence staining of pmTOR (red) was performed with cell-specific markers: neuronal nuclei (NeuN, green) for neurons, glial fibrillary acidic protein (GFAP, green) for astrocytes and Ionized calcium binding adapter molecule 1 (IBA1, green) for microglia. The pmTOR was distributed in the spinal dorsal horn **(A,B)**, and was co-localized with neurons **(F)**, and microglia **(N)**, but not with astrocytes. Scale bar-200 μm **(A,B)**; 50 μm **(C–N)**.

Immunofluorescence staining of spinal pmTOR was performed on samples taken 4 h after plantar incision. Expression of pmTOR was predominantly distributed in the spinal dorsal horn ipsilateral of the plantar incision (Figures 4A,B). Triple immunofluorescence staining of pmTOR (red) with cell-specific markers was performed as follows: neuronal nuclei (NeuN, green) for neurons, glial fibrillary acidic protein (GFAP, green) for astrocytes and Ionized calcium binding adapter molecule 1 (IBA1, green) for microglia. The pmTOR was co-localized with neurons (Figures 4C–F), and microglia (Figures 4K–N), but not with astrocytes (Figures 4G–J).

### The Activation of Spinal mTOR Induced by Plantar Incision Was Dependent on Akt

**FIGURE 5:**
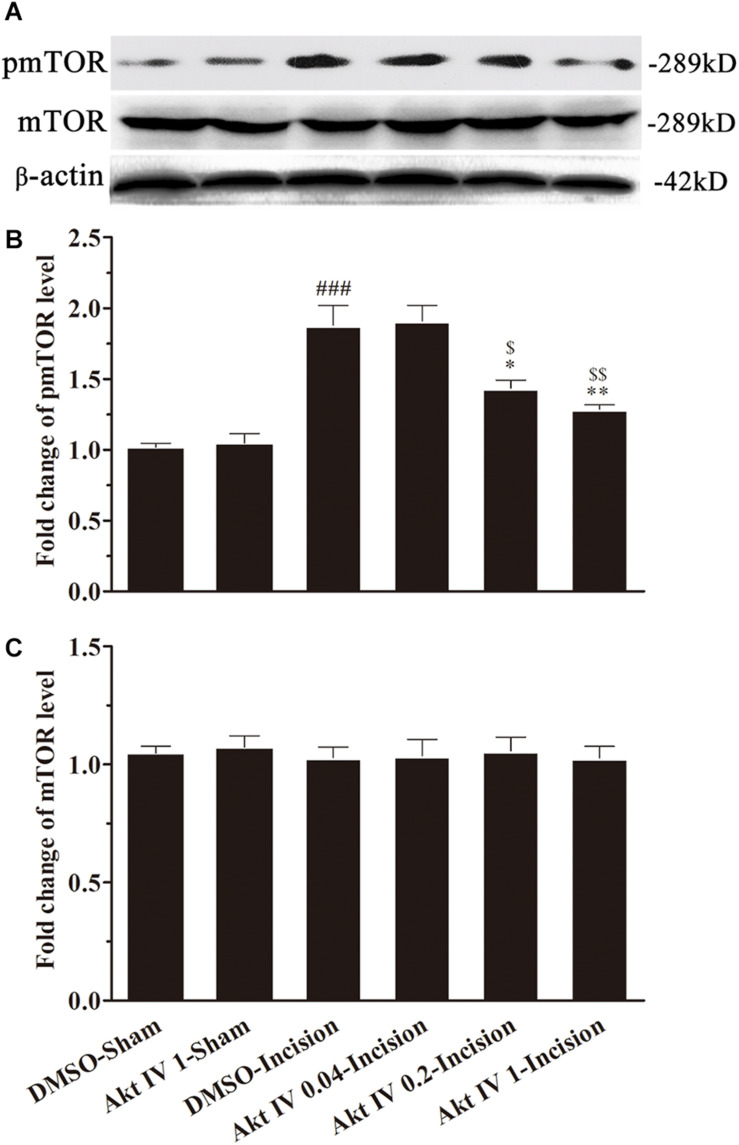
Akt IV inhibited the activation of spinal mTOR induced by plantar incision. Inhibitors of Akt, Akt IV (0.04, 0.2, and 1 μg in 5 μl of 5% DMSO) or vehicle (5 μl of 5% DMSO) were intrathecally injected 30 min before plantar incision. The expression pmTOR and mTOR protein was assayed 4 h after plantar incision by western blot. Pre-treatment with Akt IV dose-dependently prevented the up-regulation of spinal pmTOR induced by plantar incision. The representative bands **(A)** for the expression of pmTOR and mTOR at different time points after plantar incision and the quantitative data for the expression of pmTOR **(B)** and mTOR **(C)** are shown. ^###^*P*<0.001, compared with DMSO -Sham group; *P<0.05, **P<0.01, compared with DMSO-Incision group; ^$^*P*<0.05, ^$$^*P*<0.01, compared with Akt IV 0.04-Incision group. One-way ANOVA followed by Bonferroni’s Multiple Comparison Test. *n* = 4. Data were presented as mean ± SEM.

**FIGURE 6:**
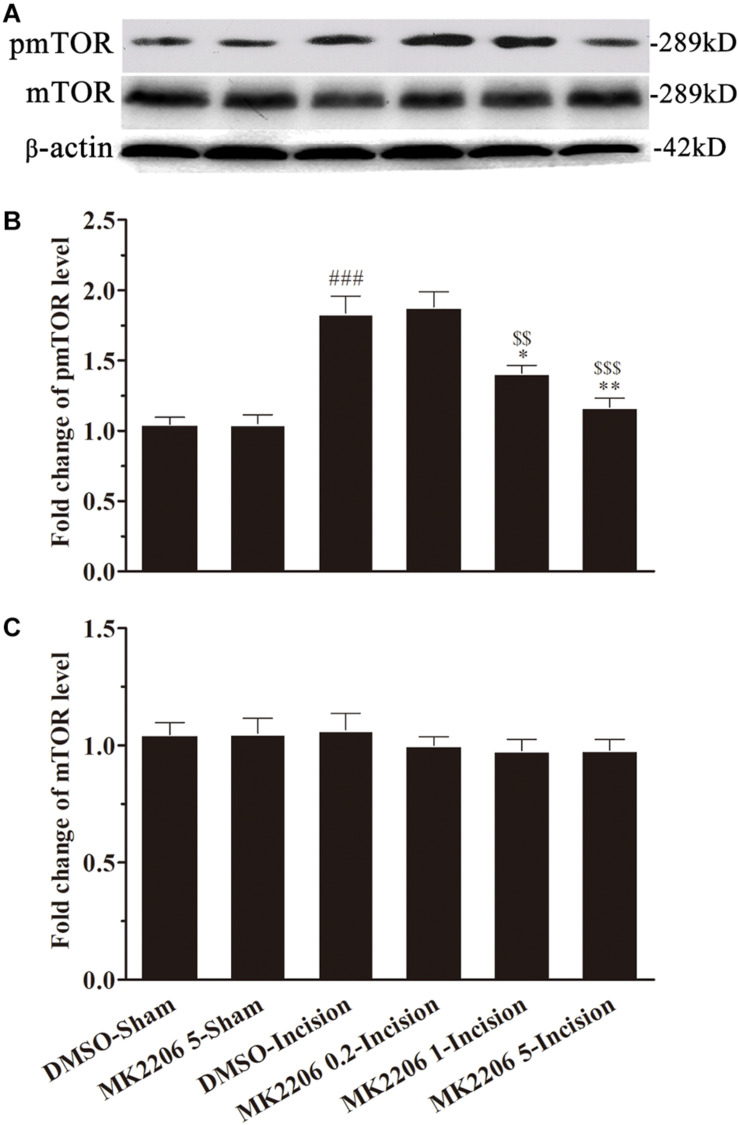
MK2206 inhibited the activat.ion of spinal mTOR induced by plantar incision. Inhibitors of Akt, MK2206 (0.2, 1, and 5 μg in 5 μl of 5% DMSO) or vehicle (5% DMSO 5 μl) were intrathecally injected 30 min before plantar incision. The expression pmTOR and mTOR protein was assayed 4 h after plantar incision by western blot. Pre-treatment with MK2206 dose-dependently prevented the up-regulation of spinal pmTOR induced by plantar incision. The representative bands **(A)** for pmTOR and mTOR expression at different time point after plantar incision and the quantitative data for pmTOR **(B)** and mTOR **(C)** expression are shown. ^###^*P*<0.001, compared with DMSO-Sham group; ^∗^*P*<0.05, ^∗∗^*P*<0.01, compared with DMSO-Incision group; ^$$^*P*<0.01, ^$$$^*P*<0.001, compared with MK2206 0.2-Incision group. One-way ANOVA followed by Bonferroni’s Multiple Comparison Test. *n* = 4. Data were presented as mean ± SEM.

In order to explore the role of Akt in the activation of mTOR induced by plantar incision, different doses of two Akt inhibitors: Akt IV (0.04, 0.2, and 1 μg in 5 μl of 5% DMSO) or MK2206 (0.2, 1, and 5 μg in 5 μl of 5% DMSO) were administered intrathecally 30 min before plantar incision. The expression of spinal pmTOR was assayed 4 h after plantar incision, using western bolt. Pre-treatment with Akt IV or MK2206 dose-dependently prevented the up-regulation of spinal pmTOR induced by plantar incision, but did not affect the expression of spinal mTOR (Figures 5, 6).

### Inhibition of Spinal mTOR Prevented the Pain-Related Behavior Induced by Plantar Incision

**FIGURE 7:**
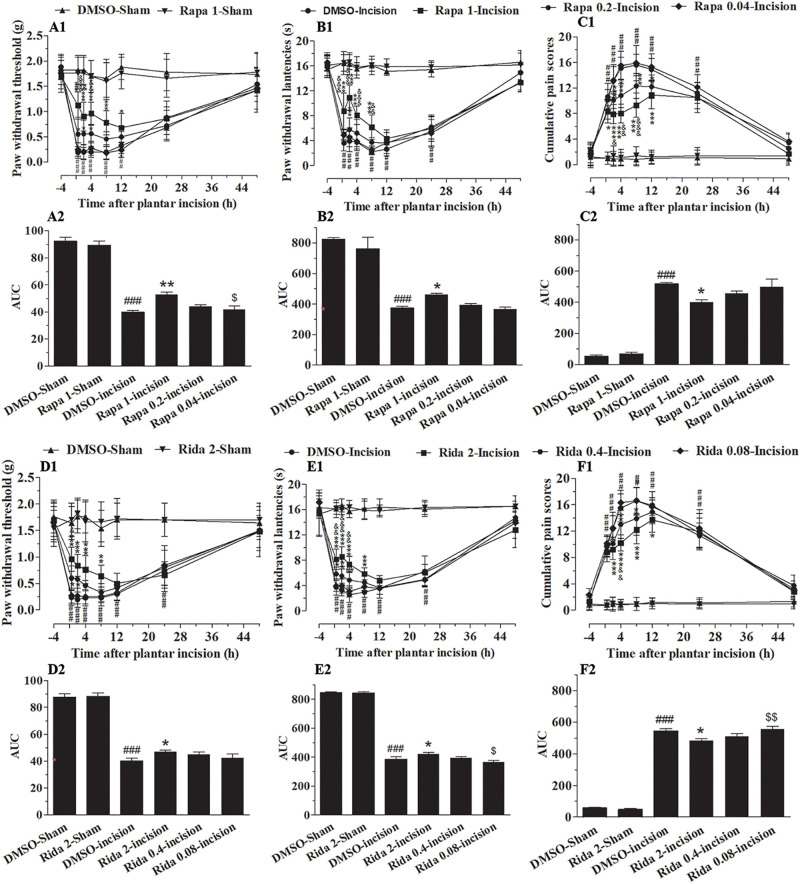
Inhibition of spinal mTOR prevented the pain-related behavior induced by plantar incision. Two selective inhibitors of mTOR, Rapa (0.04, 0.2, and 1 μg in 5 μl of 5% DMSO), Rida (0.08, 0.4, and 2 μg in 5% DMSO 5 μl), or vehicle (5% DMSO 5 μl) were administered intrathecally 30 min before plantar incision. Paw withdrawal threshold (PWT) to mechanical stimuli, paw withdrawal latency (PWL) to radiant heat and cumulative pain scores (CPS) were recorded at −4, 0.5, 2, 4, 8, 12, 24, and 48 h after plantar incision. Pre-treatment with various doses of Rapa **(A1–C1)** or Rida **(D1–F1)** attenuated the decrease of PWT **(A1,D1)** and PWL **(B1,E1)**, or the up-increase in CPS **(C1,F1)** induced by plantar incision. The calculated area under the curve (AUC) in the PWL **(B2,E2)** and PWT **(A2,D2)** tests was significantly increased in incised groups given either Rapa (1 μg) or Rida (2 μg) and was significantly decreased in the same groups in CPS **(C2,F2)**.^###^*P*<0.001, compared with DMSO -Sham group; *P<0.05, **P<0.01, ***P<0.001, compared with DMSO-Incision group;^&^*P*<0.05, ^&⁣&^*P*<0.01, ^&⁣&⁣&^*P*<0.001, compared with Rapa 0.2-Incision group or Rida 0.4-Incision group; ^$^*P*<0.05, ^$$^*P*< 0.01, compared with Rapa 1-Incision group or Rida 2-Incision group. **(A1–F1)** Two-way repeated measure ANOVA followed by Bonferroni’s posttest, “Drug” was treated as a “between” subject factor, “Time” was treated as “within subjects” factor. **(A1)** Drug: *F*(7, 315) = 115.36, *P*< 0.0001; Time: *F*(4, 315) = 419.67, *P*< 0.0001. **(B1)** Drug: *F*(7, 315) = 301.77, *P*< 0.0001; Time: *F*(4, 315) = 612.98, *P*< 0.0001. **(C1)** Drug: *F*(7, 315) = 231.87, *P*< 0.0001; Time: *F*(4, 315) = 519.38, *P*< 0.0001. **(D1)** Drug: *F*(7, 315) = 120.06, *P*< 0.0001; Time: *F*(4, 315) = 198.13, *P*< 0.0001. **(E1)** Drug: *F*(7, 315) = 331.86, *P*< 0.0001; Time: *F*(4, 315) = 809.17, *P*< 0.0001. **(F1)** Drug: *F*(7, 315) = 323.01, *P*< 0.0001; Time: *F*(4, 315) = 401.37, *P*< 0.0001. **(A2–F2)** One-way ANOVA followed by Bonferroni’s Multiple Comparison Test. *n* = 10. Data were presented as mean ± SEM.

To more specifically define the involvement of spinal mTOR on plantar-induced pain-related behavior, we examined the effect of intrathecal delivery of inhibitors of mTOR in plantar incision-induced pain-related behavior. Two selective inhibitors of mTOR, Rapa and Rida were administered intrathecally 30 min before plantar incision. Administration of Rapa (0.04, 0.2, and 1 μg in 5 μl of 5% DMSO) or Rida (0.08, 0.4, and 2 μg in 5 μl of 5% DMSO) attenuated mechanical allodynia and thermal hyperalgesia in a dose-dependent manner (Figure 7). Compared to the DMSO-Incision group, PWL in the incision groups was significantly elevated by Rapa (Figure 7B1: 0.2 μg at 2 h, 1 μg at 0.5–8 h) and by Rida (Figure 7E1: 0.4 μg at 0.5–4 h, 2 μg at 0.5–8 h) (*P*< 0.05, 0.01, or 0.001). Compared to the DMSO-Incision group, PWT in the incision was significantly elevated by Rapa (Figure 7A1: 0.2 μg, at 0.5–4 h; 1 μg at 0.5–12 h) or by Rida (Figure 7D1: 0.4 μg at 0.5–2 h; 2 μg at 0.5–8 h) (*P*< 0.05, 0.01, or 0.001). Additionally, compared to the DMSO-Incision group, the cumulative pain scores (CPS) significantly decreased in the incision groups after Rapa (Figure 7C1: 0.2 μg at 0.5–12 h; 1.0 μg at 0.5–12 h) or Rida (Figure 7F1: 0.4 μg at 2–8 h; 2 μg at 2–12 h) (*P*< 0.05, 0.01, or 0.001). In these behavioral tests, intrathecal Rapa or Rida did not alter the PWL, PWT, or CPS in sham-surgerized groups. Further, intrathecal vehicle (5 μl of 5% DMSO) did not affect pain-related behavior at any time points. The calculated area under the curve (AUC) in the PWL (Figures 7B2,E2) and PWT (Figures 7A2,D2) tests (*P*< 0.05 or *P*< 0.01), was significantly increased in incised groups given either Rapa (1 μg) or Rida (2 μg) and was significantly decreased in the same groups in CPS (Figures 7C2,F2, *P* < 0.05).

### Inhibition of Spinal mTOR Attenuated the Up-Regulation of Spinal Fos Protein Induced by Plantar Incision

**FIGURE 8:**
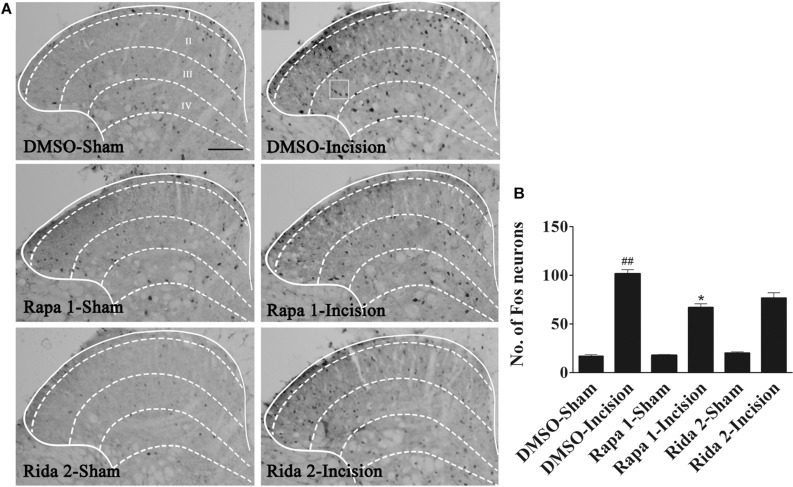
Blocking of spinal mTOR prevented the up-regulation of spinal Fos protein expression induced by plantar incision. Inhibitors of mTOR, Rapa (1 μg in 5 μl of 5% DMSO), Rida (2 μg in 5 μl of 5% DMSO), or vehicle (5% DMSO 5 μl) were intrathecally injected 30 min before plantar incision. Fos protein expression was assayed 1 h after plantar incision. **(A)** Representative immunohistochemical staining of spinal Fos protein expression. **(B)** Quantitative data of spinal Fos protein expression.^##^*P*<0.01, compared with DMSO -Sham group; *P< 0.05, compared with DMSO-Incision group. One-way ANOVA followed by Bonferroni’s Multiple Comparison Test. *n* = 6, scale bar = 200 μm.

In order to clarify the analgesic effect of mTOR inhibitors, Rapa (1 μg in 5 μl of 5% DMSO) and Rida (2 μg in 5 μl of 5% DMSO) or 5% DMSO (5 μl) was intrathecally injected 30 min before plantar incision. The expression of Fos protein was assayed by using immunohistochemistry in samples taken 1 h after plantar incision. Compared with the DMSO-Sham group, plantar incision produced a significant increase of Fos protein in the ipsilateral spinal dorsal horn in DMSO-Incision group (Figures 8A,B). Compared with DMSO-Incision group, Rapa or Rida significantly attenuated the increase of Fos protein in incised groups given either Rapa (1 μg) or Rida (2 μg) (*P*< 0.05). Fos expression was not altered by Intrathecal vehicle (5% DMSO), Rapa or Rida in sham surgerized groups.

### Interactions Between pmTOR and Phosphorylated Akt (pAkt) in the Spinal Cord After Plantar Incision

**FIGURE 9:**
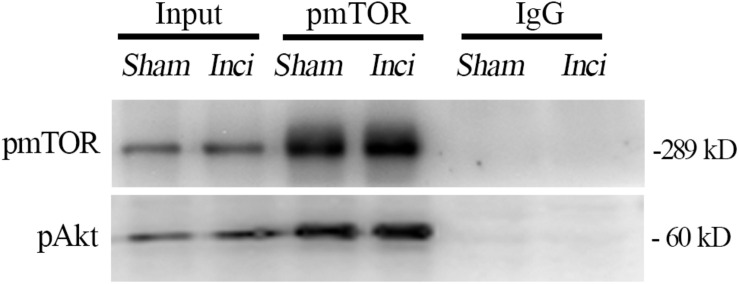
Interactions between pmTOR and pAkt in the spinal cord after plantar incision. The interaction between pmTOR and pAkt in the spinal cord was determined by co-immunoprecipitation analysis. Spinal cord homogenates (4 h after plantar incision or sham operation) were immunoprecipitated with pmTOR antibody, and immunoblotted with pAkt antibody. *n* = 4.

The interaction between pmTOR and pAkt in the spinal cord was determined by co-immunoprecipitation analysis done on samples taken 4 h after plantar incision. The results showed that pAkt was co-immunoprecipitated by anti-pmTOR antibody in the spinal cord 4 h after plantar incision. Additionally, pmTOR and pAkt complex was seen in sham-surgerized mice (Figure 9), indicating that this complex exists in the spinal cord and is also linked to plantar incision pain.

## Discussion

Management of POP is still a major issue. Many mechanisms have been investigated. Most recent research focused on opioid-induced hyperalgesia and endogenous descending inhibition (Pogatzki-Zahn et al., 2018). In this study, we found that plantar incision in mice enhanced responsiveness to mechanical and thermal stimuli, which are indicative of primary mechanical allodynia and thermal hyperalgesia. Plantar incision increased the expression of spinal pAkt and pmTOR. Primary mechanical allodynia and heat hyperalgesia after plantar incision were blocked by inhibiting the Akt-mTOR pathway. Our observations demonstrate that Akt-mTOR pathway contributes to POP in the spinal dorsal horn.

Akt was discovered as a major regulator of cell cycle in the 1990s (Brazil et al., 2004). Spinal cord expression of Akt and its downstream effectors suggests their involvement in the transmission and modulation of noxious information (Sun et al., 2006, 2007; Xu et al., 2007, 2011; Guedes et al., 2008; Choi et al., 2012; Duan et al., 2012; Kay et al., 2013; Li et al., 2013; Hung et al., 2014; Xu J.T. et al., 2014; Zhang et al., 2014, 2019; Guan et al., 2015; Kondo et al., 2018; Liu et al., 2019; Xing et al., 2020). Our data show that Akt was quickly activated by plantar incision in mice, and the pain-related behaviors were attenuated by blocking the phosphorylation of Akt. Protein phosphorylation is one of the most important mechanistic processes used in the nervous system for the efficacy and specificity of neurotransmitter release from the presynaptic terminal (Greengard et al., 1993; Greengard, 2001). We conclude that the phosphorylation of Akt may serve as a neuro-modulator in plantar incision-induced synaptic function and plasticity. The results presented herein, support targeting Akt for developing new analgesic pharmacotherapies, which would ameliorate acute POP, and potentially inflammatory and chronic pain. Three Akt isoforms have been identified: Akt1, Akt2, and Akt3 (Emamian, 2012). Of the three isoforms, Akt2 in the DRG mediates oxaliplatin-induced neuropathic pain in mouse models (Jiang Z. et al., 2016). The regulatory mechanism of miR-15a, miR-145, and miR-150 in chronic constriction injury- induced neuropathic pain requires inhibiting the expression of Akt3 (Shi et al., 2018; Cai et al., 2019, 2020; You et al., 2019). Unfortunately, little is known about the specific role of Akt1 in pain processes. In future studies, we would like to do a more in-depth characterization of how the different Akt isoforms are involved in POP.

The mammalian target of rapamycin kinase (mTOR), a downstream signaling molecule of Akt, was first described as TOR1 and TOR2 in *Saccharomyces cerevisiae* mutants (Heitman et al., 1991). Studies show that blocking mTOR at the peripheral level, spinal level or supraspinal level alleviated pain-related behaviors associated with inflammation, neuropathic pain, or bone cancer pain (Jimenez-Diaz et al., 2008; Asante et al., 2009; Geranton et al., 2009; Obara et al., 2011; Lyu et al., 2013; Zhang et al., 2013, 2019; Cui et al., 2014; [Bibr B23][Bibr B28]; Zarpelon et al., 2016; Kwon et al., 2017; Kondo et al., 2018; Liu et al., 2019; Kim et al., 2020; Choi et al., 2020; Xing et al., 2020). Our results indicate that plantar incision increased the expression of spinal pmTOR in a time-dependent manner. Xu et al. (2010) indicated that acute nociceptive transmission was not medicated by spinal mTOR. In their research, acute nociceptive transmission was induced in naïve rats by directly applying high-intensity radiant heat and von Frey monofilaments to the plantar sides of hind paws under normal condition. Our results showed that the decrease of both PWT and PWL accompanied by the increase of spinal Fos expression and CPS, induced by plantar incision, were significantly inhibited by Rapa or Rida. We conclude that the phosphorylation of spinal mTOR may service as a neuro-modulator during the processes of synaptic function and plasticity induced by plantar incision, even the plantar incision may be one kinds of acute nociception.

Additionally, mTOR has been reported to regulate activity of microglia and astrocytes (Dello et al., 2013; Chen et al., 2015). Our results showed that pmTOR was predominantly distributed in the spinal dorsal horn ipsilateral of the plantar incision (Figures 4A,B), and was co-localized with neurons (Figures 4C–F) and microglia (Figures 4K–N), but not with astrocytes (Figures 4G–J). These data suggest that pmTOR may regulate microglia activity after plantar incision. These contradictory findings may result from different species and models. The specific mechanisms leading to allodynia and hyperalgesia during operation are unclear. A number of studies have confirmed that the relationship between immune cells and neurons and between glial cells and neurons, modulates various aspect of pain including: signal transduction, transmission, perception, regulation, and the conversion of acute pain to chronic pain (Kurrikoff et al., 2004; Tanga et al., 2005; Koks et al., 2008). For example, microglial Toll-like receptor 4 (TLR4) contributes to the initiation of CNS neuroimmune activation in neuropathic pain (Tanga et al., 2005; Koks et al., 2008). Similarly, some neuropeptides such as cholecystokinin regulate neuropathic pain (Kurrikoff et al., 2004; Koks et al., 2008). Whether POP is regulated by an antagonistic interaction between Akt-mTOR pathway, cholecystokinin systems, and the immune system, needs further study.

The Akt-mTOR signaling cascade contributes to some physiological and pathological processes, such as morphine tolerance, hyperalgesia, reconsolidation of cocaine contextual reward memory, protection against stroke, and angiogenesis, just to name a few (Shi et al., 2014; Xiong et al., 2014; Xu J.T. et al., 2014). Akt plays a role in synaptic plasticity and memory through the regulation of mTOR via phosphorylation and inhibition of tuberous sclerosis complex 2 (TSC2) (Klann and Dever, 2004; Costa-Mattioli et al., 2009). Co-immunoprecipitation data showed that pmTOR interacted with pAkt in the spinal cord, under plantar incision condition or normal condition (Figure 9). Additionally, the pretreatment of Akt inhibitors (MK2206 or Akt inhibitor IV) prevented the phosphorylation of mTOR (Figures 5, 6). The data presented here suggest that after plantar incision, mTOR may be the downstream target of Akt at the spinal level. Future work still needs to detail the particular mechanisms by which Akt elicits mTOR activity.

There are some limitations in this study. First, only male mice were explored. Sex influences on pain or analgesia has been identified in preclinical and clinical studies (Xu et al., 2019), the conclusion of this study cannot be directly extended to female rodents. Second, the inhibitors have a wide range of action, which does not exclude the possibility of acting on other targets.

## Conclusion

In conclusion, the results presented here provided evidence that the Akt-mTOR pathway mediates POP in the spinal dorsal horn. Additionally, these results indicate that the Akt-mTOR pathway may be a promising target for developing new analgesics, which would ameliorate acute POP, as well as inflammatory pain and chronic pain.

## Data Availability Statement

All datasets generated for this study are included in the article/supplementary material.

## Ethics Statement

The animal study was reviewed and approved by the People’s Hospital of Guangxi Zhuang Autonomous Region, Nanning; No. 2018-06.

## Author Contributions

BX, LM, and X-HG designed the experiments and drafted the manuscript. S-SL, CM, and JW performed the mice model of POP, intrathecal injections, and behavior testing. CM and C-ML performed immunohistochemistry, western blotting, and co-immunoprecipitation. Z-YJ, A-LH, and Q-BC performed the statistics. All authors agreed to be accountable for the content of the work, and approved the final manuscript.

## Conflict of Interest

The authors declare that the research was conducted in the absence of any commercial or financial relationships that could be construed as a potential conflict of interest.
